# Comparative efficacy of phosphorous supplements with phosphate solubilizing bacteria for optimizing wheat yield in calcareous soils

**DOI:** 10.1038/s41598-022-16035-3

**Published:** 2022-07-14

**Authors:** Muhammad Adnan, Shah Fahad, Muhammad Hamzah Saleem, Baber Ali, Maria Mussart, Rafi Ullah, Amanullah Jr, Muhammad Arif, Manzoor Ahmad, Wajid Ali Shah, Muhammad Romman, Fazli Wahid, Depeng Wang, Shah Saud, Ke Liu, Matthew Tom Harrison, Chao Wu, Subhan Danish, Rahul Datta, Crina Carmen Muresan, Romina Alina Marc

**Affiliations:** 1 Department of Agriculture, The University of Swabi, Swabi, 23561 Pakistan; 2 Hainan Key Laboratory for Sustainable Utilization of Tropical Bioresource, College of Tropical Crops, Hainan University, Haikou, 570228 Hainan China; 3 Department of Agronomy, The University of Haripur, Haripur, 22620 Pakistan; 4 College of Plant Science and Technology, Huazhong Agricultural University, Wuhan, 430070 China; 5 Department of Plant Sciences, Quaid-I-Azam University, Islamabad, 45320 Pakistan; 6 Department of Soil and Environmental Sciences, The University of Agriculture, Peshawar, Pakistan; 7 Department of Agronomy, The University of Agriculture, Peshawar, Pakistan; 8 Department of Agriculture, Bacha Khan University, Charsadda, Pakistan; 9 Department of Botany, University of Chitral, Chitral, Pakistan; 10 College of Life Science, Linyi University, Linyi, 276000 Shandong China; 11 Hubei Collaborative Innovation Center for Grain Industry/Agriculture College, Yangtze University, Jingzhou, 434025 Hubei China; 12 Tasmanian Institute of Agriculture, University of Tasmania, Burnie, TAS 7250 Australia; 13 Guangxi Key Laboratory of Functional Phytochemicals Research and Utilization, Guangxi Institute of Botany, Guangxi Zhuang Autonomous Region and Chinese Academy of Sciences, Guilin, 541006 China; 14 Department of Soil Science, Faculty of Agricultural Sciences and Technology, Bahauddin Zakariya University, Multan, 60800 Pakistan; 15 Department of Geology and Pedology, Faculty of Forestry and Wood Technology, Mendel University in Brno, 61300 Brno, Czech Republic; 16 Food Engineering Department, Faculty of Food Science and Technology, University of Agricultural Science and Veterinary Medicine Cluj-Napoca, 3-5 Calea Mănă ¸stur Street, 400372 Cluj-Napoca, Romania

**Keywords:** Ecology, Plant sciences, Environmental sciences

## Abstract

Phosphorus (P) deficiency is the main hurdle in achieving sustainable crop production ps especially in calcareous soils. Using bio-fertilizers like phosphate solubilizing bacteria (PSB) could be a useful approach for sustainable P management as they improve P availability in soil via dissolution, desorption and mineralization reactions. In addition, application of organic amendments with PSB could further ameliorate soil conditions for sustainable management of immobilized nutrients in calcarious soils. Therefore, we performed pot experiment to study the role of PSB in nullifying antagonistic effects of liming (4.78, 10, 15 and 20%) on P availability from poultry manure (PM), farm yard manure (FYM), single super phosphate (SSP) and rock phosphate (RP) in alkaline soils. PSB inoculation improved wheat growth, P availability and stimulated soil acidification over control regardless of P sources and lime levels. Soil calcification adversely affected plant growth, P nutrition, induced soil salinity and alkalinity, however, PSB and manures application potentially nullified such harmful effects over mentioned traits. Individually, organic sources were superior than mineral sources however, the performance of mineral fertilizers with PSB was at par to sole application of manures. Furthermore, application of RP with PSB proved as effective as sole SSP. Therefore, using PSB as bio-fertilizer has huge potential for improving P availability in calcareous soils.

## Introduction

Optimum availability of Phosphorus (P) for humans, plants and animals on sustainable basis is a fundamental prerequisite to meet the worldwide hunger by 2030^1^. Sustainable management of P at global scale is a fundamental aspect for the achievement of global food. Phosphorus performs vital functions in plant metabolism, growth and development. It cannot be manufactured by plants and has no substitute, thus management of phosphorus require sustainable measures to improve its crops use efficiency. Globally, deficiency of P is a main yield reducing nutrient next to nitrogen^2^. It also cannot be fixed from atmosphere biologically as N dose, that’s why mineralization of P in soils is a key factor that can enhanced its bioavailability for agricultural crops on sustainable basis^3^. Primary and secondary ortho-phosphate, and phosphate are the bio-available mineral forms of P in soil. These are subjected to losses due to adsorption on clay surfaces^4^, and/or through precipitation with cations like Ca^+2^ and Mg^+2^ at high pH or Fe^+2^ and Al^+3^ at low pH. Consequently, the bio-available P in the soil is as low as 0.1 mg kg^−1^^5^ and nearly, 30–40% cultivable land across the globe suffers from P deficiency. Khan et al.^6^ reported that, the reserved P in soil is sufficient to support optimum plant growth for 100 years, if it is made bio-available by certain means.

Calcareous soils are the most abundant (800 million hectares worldwide) in arid and semi-arid areas^7^. These soils contain high quantity of calcite that fixes significant quantities of P either by precipitation with Ca^+2^ and Mg^+2^ and/or by sorption on calcite surfaces (16 to 200 m^2^ g^−1^)^8^. Consequently, 90% of calcareous soils are scarce in bio-available P^9^. To fulfill plant P requirements, these soils are regularly supplemented with phosphatic fertilizers^10^. According to Goldstein^11^ almost 30 million tons (MT) of P fertilizers (worth 4 billion USD) are added to the soils throughout the world. About 20% of added P is used by the plants while the rest of 80% is lost through different processes^12^. Such losses not only rise cost of production but also cause environmental pollution^13^. Organic supplements added to calcareous soils affect soil P chemistry by forming insoluble complexes like Ca-phytates^14^. Rock phosphate (RP) which contains 17% P can be used as an economical alternative for substituting expensive chemical P fertilizers^5,15^, however, it solubility is very low in calcareous soils^16^. Thus, these circumstances have compelled the scientists to find eco-friendly and economically feasible substitutes of these sources to improve crop yield and P nutrition in P deficient soils^17^.

Using phosphate solubilizing bacteria (PSB) as an alternative to expensive mineral P fertilizers could be an environmental-friendly approach for improving crop yield in calcareous soil^15^. The PSB may increase the solubility of precipitated P like Ca_3_-(PO_4_)_2_ through the release of protons, phenolic compounds, and siderophores^18^, organic^19^ and mineral acids^20–22^. The may promote the process of biological nitrogen fixation (BNF) and may increase the availability of micro nutrients like Fe^+2^ and Zn^+2^ etc.^23^. They may also prevent P losses by immobilizing it in the presence of labile carbon^24^ and may re-add it into soil by it decomposition. They may also improve bio availability of P through liberation of extracellular enzymes^25^. The PSB may also improve crop growth and P availability in calcareous soils by the production of gibberellins^26^, cytokinins^27^, IAA, Alkaline phosphatases, hydroxyl ions and CO_2_^28^ and by H^+^ protonation^29^, anion exchange and chelation^30^. Therefore, PSB have a main role in regulating soil P cycle like sorption–desorption, dissolution–precipitation, and mineralization–immobilization process. Jalili et al.^31^ reported that, integration of PSB and PGPR could decrease the use of P fertilizer by 50% without having any adverse effect on crop yield. Many scientists observed increase in crop yield like rice^32^, maize^33^ and other cereals^34^ with PSB inoculation. Similarly, Bolan^35^ observed long term improvement in P availability and crop yield with combined use of organic and mineral P supplements than sole mineral P fertilizations in calcareous soils.

Application of PSB can reduce dependence on expensive chemical P fertilizers either by solubilizing the preserved insoluble soil P or by substituting them with environment friendly and economical natural P sources like RP. However, their density, performance and P solubilizing ability vary with different soils and production system^21^. Furthermore, limited research has been conducted on exploring the role of PSB under low organic matter containing calcareous soils. Thus, this study was conducted to explore potential of PSB for optimizing P supply and improving wheat yield in alkaline soil amended with different P supplements under different levels of lime.

## Results

### PGPR features and composition of used PSB

The peat based inoculum was composed of *Arthrobacter* (9%), *Burkholderia* (10%), *Bacillus* (16%), *Enterobacter* (3%), *Mycobacterium* (14%), *Pseudomonas* (13%), *Pantoea* (10%) and *Rhizobia* (9%) while, 16% of the colonies were unidentified (Table 1). The PSB was capable of phosphate solubilization (8.4 diameter of halo in mm), Axine (4.01 mg ml^−1^), IAA (8.3 µg ml^−1^), organic acids (10.6 g l^−1^) and siderpores (5.6 diameter of halo in mm) production (Table 2). The inoculum contained 1.75 × 10^8^ cfu PSB g^−1^ on average basis (wet weight basis).

**Table 1:** Percent bacterial composition of PSB inoculum.

Bacterial species	Percent composition
*Arthrobacter*	9
*Bacillus*	16
*Burkholderia*	10
*Enterobacter*	3
*Mycobacterium*	14
*Pantoea*	10
*Pseudomonas*	13
*Rhizobia*	9
Unidentified	16

**Table 2:** Auxine, indole acetic acid (IAA), organic acid and siderophore production, phosphate-solubilization and population by/of PSB.

PGPR characteristics	Quantity	Unit
Auxin production	4.0 ± 0.39	mg ml^−1^
IAA production	8.3 ± 0.59	µg ml^−1^
Phosphate-solubility	8.4 ± 0.52	Diameter of halo in mm
Siderophores production	5.6 ± 0.80	Diameter of halo in mm
Total organic acid	10.6 ± 0.74	g l^−1^
Population	1.75 × 10^8^	cfu g^−1^ inoculum (wet weight basis)

± Values represent SE of mean (n = 3).

### Tillers, plant height, grains per spike and 100 grains weight of wheat

Results exhibiting the effects of PSB inoculation and P sources on wheat tillers per pot (TP), plant height (PH), grains per spike (GS) and 100 grains weight (HGW) in soil with varying lime contents are presented in Table 3. Seed inoculation with PSB significantly improved TP, PH, GS and HGW by 10.7, 12.7, 10.3 and 7.0%, correspondingly, compared to without PSB pots. Similarly, among the sources poultry manure (applied @ of 45 mg P_2_O_5_ kg^−1^) produced significantly higher TP (2.29), PH (54.9 cm), GS (39.4) and HWG (4.3 g) which were statistically at par with farmyard manure (FYM) except HGW where PM performed better than FYM. The minimum values of these tested traits were noted where RP was used however, its performance was statistically similar to SSP for all traits except PH where SSP (47.9 cm) produced taller plants than RP (42.9 cm) as presented in Table 3. Liming adversely affected all the yield attributes. With increasing lime content a significant decline of 10.9, 23.7 and 39% was observed in TP, 5.3, 16.2 and 31.3% in PH, 5.6, 11 and 21.4% in GS and 3.3, 7.5 and 16.6% in HGW at 10, 15 and 20% lime over control (4.78%), respectively. However, PH responded statistically similar to control and 10% lime.

**Table 3:** Impact of PSB inoculation and P sources on wheat tillers per pot, plant height, grains per spike and 100 grains weight in soil with varying lime content.

Inoculation	Tillers plant^−1^	Plant height (cm)	Grains spike^−1^	100 grains weight (g)
Without PSB	1.84	46.52	34.71	3.85
With PSB	2.03	52.43	38.29	4.12
LSD (α = 0.05)	0.096	2.25	1.115	0.077
**P sources**				
SSP	1.64b	47.88b	34.95b	3.75c
Rock phosphate	1.57b	42.87c	33.83b	3.79c
FYM	2.25a	52.25a	37.83a	4.07b
PM	2.29a	54.91a	39.37a	4.30a
LSD (α = 0.05)	0.136	3.195	1.577	0.109
**Lime (%)**				
Control (4.78%)	2.38a	57.00a	40.33a	4.27a
10	2.12b	54.00a	38.08b	4.13b
15	1.81c	47.75b	35.87c	3.95c
20	1.45d	39.16c	31.70d	3.56d
LSD (α = 0.05)	0.136	3.195	1.577	0.109
**Interaction**				
L × I	ns	Figure 1	ns	Figure 2
L × PS	Figure 3	Figure 4	ns	ns
I × PS	ns	ns	Figure 5	ns
L × I × PS	ns	ns	ns	ns
CV	12.21	11.20	7.49	4.76

PSB, LSD, SSP, FYM, PM and ns denote phosphate solubilizing bacteria, least significant difference, single super phosphate, farmyard manure, poultry manure and non-significant interaction, respectively.

Means sharing letter in each category are statistically at par at α ≤ 0.05.

**Figure 1:**
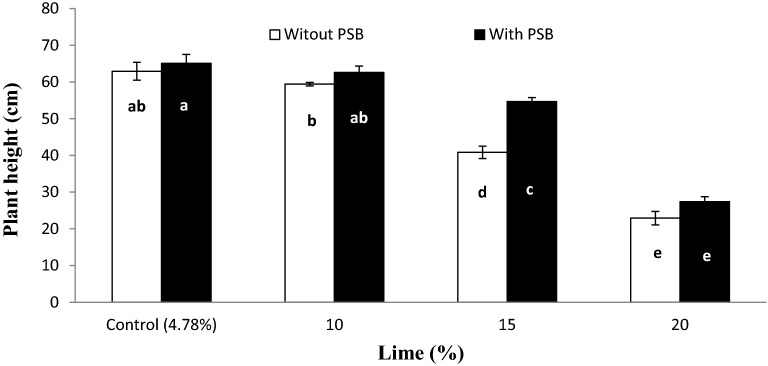
Interactive effect of lime and P sources on plant height per pot. Graph bars having different letters are significantly different at α = 0.05. Errors bar represent standard error for the mean of three values.

**Figure 2:**
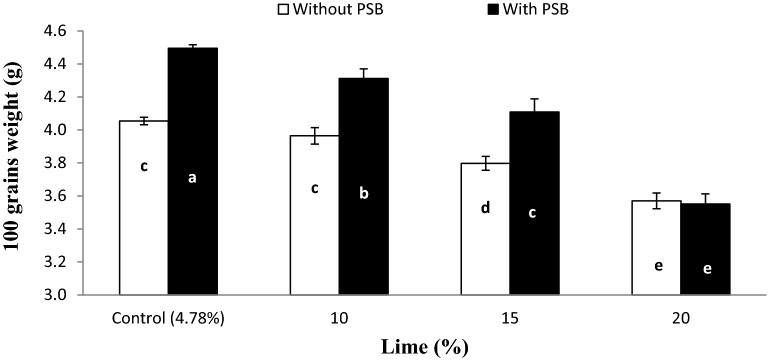
Interactive effect of lime and PSB on wheat 100 grains weight. Graph bars having different letters are significantly different at α = 0.05. Errors bar represent standard error for the mean of three values.

The interactive effect of lime × inoculum (L × I) was significant for PH (Fig. 1) and HGW (Fig. 2). The PH gradually decreased with increasing lime content from 10 to 20% both in inoculated and un-inoculated pots while, pots treated with 10% lime produced taller plants which were statistically comparable to control (4.78%) lime both with and without PSB inoculation. Inoculated and un-inoculated plants performed statistically similar at all lime contents except 15% where PSB inoculation significantly increased PH over un-inoculated. Inoculated treatments produced heavier grains than un-inoculated soil at all lime contents except 20% where inoculation didn’t affect the 100 grains weight. Furthermore, 15% lime with PSB and 10% lime without PSB produced 100 grains with similar weight (Fig. 2).

**Figure 3:**
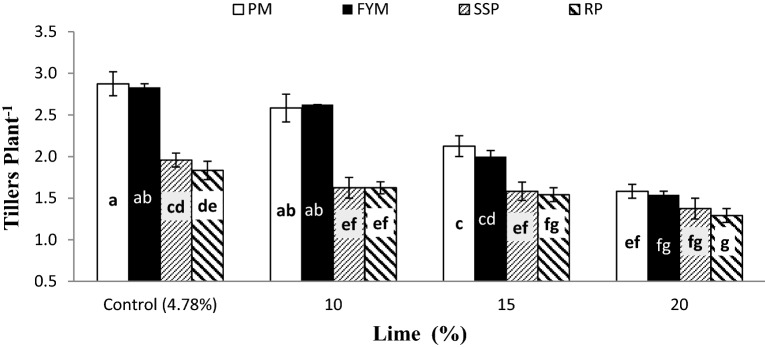
Interactive effect of lime and PSB on wheat tillers per plant. Graph bars having different letters are significantly different at α = 0.05. Errors bar represent standard error for the mean of three values.

**Figure 4:**
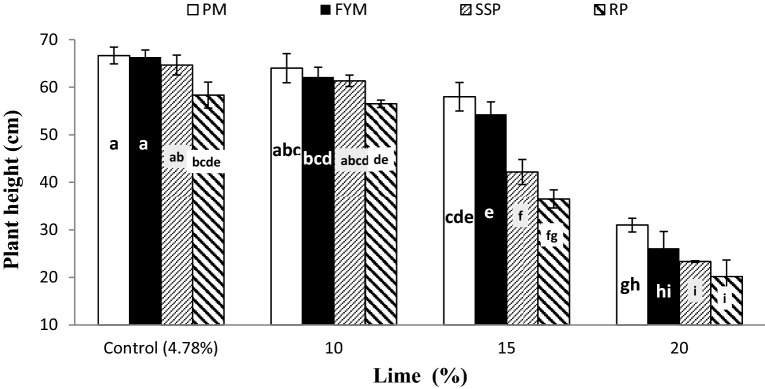
Interactive effect of lime and P sources on plant height. Graph bars having different letters are significantly different at α = 0.05. Errors bar represent standard error for the mean of three values.

Data regarding the significant interaction of lime × P sources (L × PS) for TP and PH are presented in Figs. 3 and 4, respectively. Organic sources (PM and FYM) produced significantly higher TP than mineral sources (SSP and RP) at all levels of lime except 20%. At 20% lime PM, FYM and SSP showed similar effect on TP however, RP performance was notably poor than PM and at par to FYM and SSP. Likewise, the TP responded alike to PM and FYM at 20% lime and SSP and RP with 15% lime. Non-significant intra source difference were observed on both the organic (between PM and FYM) and mineral (between SSP and RP) P supplements at all lime contents. Liming negatively affected PH at all P sources, however its adverse effect was more prominent in mineral sources than organic sources at 15 and 20% lime (Fig. 4). Soil calcification up to 10% did not show any adverse effect on PH over control (4.78%), but addition of lime beyond 10% resulted in dwarf plants both at organic and mineral P application. Poultry manure acted more potentially for harmonizing the harmful effect of lime than the other sources at all lime content however its performance was similar to FYM and SSP at control and 10% lime, and FYM at 15 and 20% lime. Phosphorus applied as RP produced shorter plants at all lime contents. Furthermore, similar stature plants were observed both at 20% lime + organic sources (PM and FYM) and 15% lime + mineral P sources (SSP and RP) and 15% lime with organic sources and 10% lime with mineral P sources.

**Figure 5:**
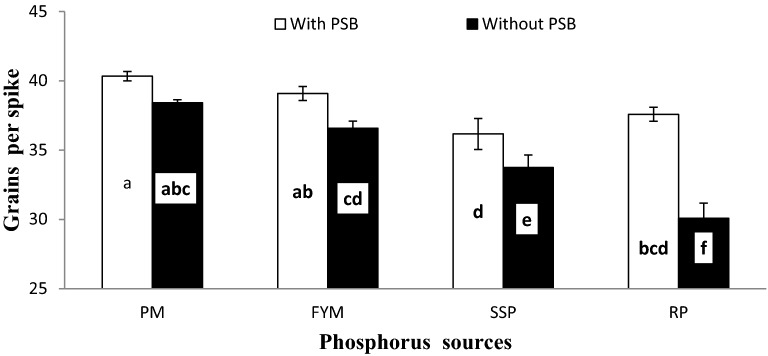
Interactive effect of P sources and PSB on wheat grains per spike. Graph bars having different letters are significantly different at α = 0.05. Errors bar represent standard error for the mean of three values.

Significant (*p* ≤ 0.01) interactive effect of inoculation and P sources (I × PS) for grains per spike (GS) showed that inoculation produced denser spike than un-inoculated plants at all P sources except PM (Fig. 5). Generally, organic sources produced significantly higher GS than mineral sources. Phosphorus applied as PM along with PSB produced statistically at par GS to PM without PSB and FYM with PSB which was considerably higher than FYM without PSB. The SSP and RP with PSB responded similar with respect to GS however, the performance of SSP alone (without PSB) was potentially better than RP without inoculation. Furthermore, RP with PSB produced statistically more filled spike than sole SSP.

### Root dry weight, grain and biological of wheat

Grain yield (GY), biological yield (BY) and root dry weight (RDW) of wheat were significantly influenced by PSB inoculation, soil calcification and phosphorus sources (Table 4). Inoculation with PSB inoculation improved GY, BY and RDW by 14.1, 16.3 and 8.1% over un-inoculated control. Generally, organic sources (PM and FYM) performed well than mineral sources (SSP and RP). Poultry manure produced maximum GY, BY and RDW which was significantly higher than FYM for GY and BY and at par with FYM for RDW. Similarly, poor performance was observed for RP in all traits which was comparable to SSP in case of GY and RDW and significantly lower than SSP for BY. On the basis of performance, the P sources could be ranked as PM ≥ FYM > SSP ≥ RP. The GY, BY and RDW gradually decreased with increasing lime content from control to 20%. With increasing level of lime wheat grain, biological and root dry weight were significantly decreased (Table 4).

**Table 4:** Wheat root dry weight, harvest index, grain, biological and straw yield as influenced by PSB inoculation, soil calcification and phosphorus application from different sources.

Inoculation	Grain yield (g) pot^−1^	Biological yield (g) pot^−1^	Root dry weight (g)
Without PSB	6.40	18.07	3.70
With PSB	7.30	21.02	4.00
LSD (α = 0.05)	0.249	0.467	0.150
**P sources**			
SSP	6.28c	17.55c	3.47b
Rock phosphate	5.88cd	16.71d	3.48b
FYM	7.39b	21.46b	4.12a
PM	7.86a	22.47a	4.30a
LSD (α = 0.05)	0.353	0.660	0.212
**Lime (%)**			
Control (4.78%)	9.29a	22.40a	4.46a
10	7.68b	20.86b	4.09b
15	6.03c	19.80c	3.70c
20	4.40d	15.13d	3.14d
LSD (α = 0.05)	0.353	0.660	0.212
**Interaction**			
L × I	Figure 6	ns	ns
L × PS	Figure 7	ns	ns
I × PS	ns	Figure 8	Figure 9
L × I × PS	ns	ns	ns
CV	8.93	5.86	9.59

PSB, LSD, SSP, FYM, PM and ns denote phosphate solubilizing bacteria, least significant difference, single super phosphate, farmyard manure, poultry manure and non-significant interaction, respectively.

Means with different letter in each column are significantly different at *p* ≤ 0.05.

**Figure 6:**
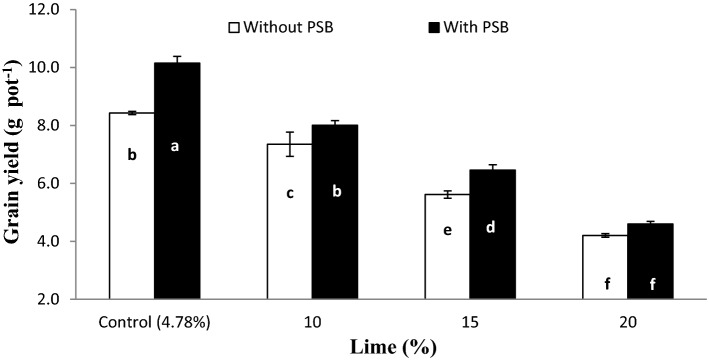
Interactive effect of lime and PSB on grain yield (g pot^−1^). Graph bars having different letters are significantly different at α = 0.05. Errors bar represent standard error for the mean of three values.

The interaction of lime × inoculum (L × I) was significant for GY at *p* ≤ 0.01 (Fig. 6). Wheat grain yield decreased with addition of lime at each lime level both in inoculated and un-inoculated pots. Similarly, PSB inoculation significantly improved GY over un-inoculated pots at all lime contents except 20% lime. Furthermore, it was also evident that 10% lime with inoculation resulted similar quantity of grains as control lime without inoculation. Highest GY was noticed for control lime with inoculation while the lowest was observed at both 20% lime with and without inoculation.

**Figure 7:**
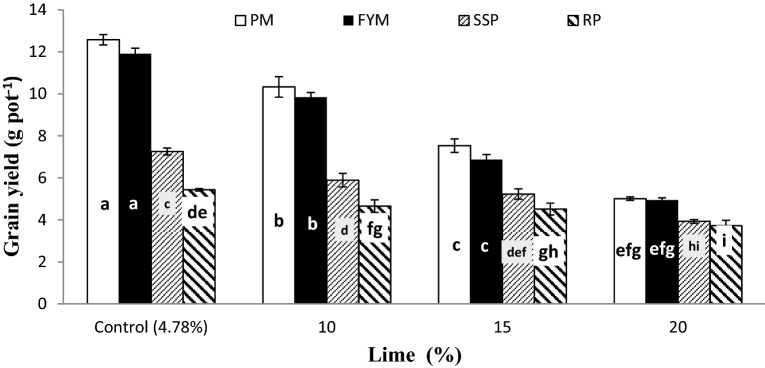
Interactive effect of lime and P sources on grain yield (g pot^−1^). Graph bars having different letters are significantly different at α = 0.05. Errors bar represent standard error for the mean of three values.

**Figure 8:**
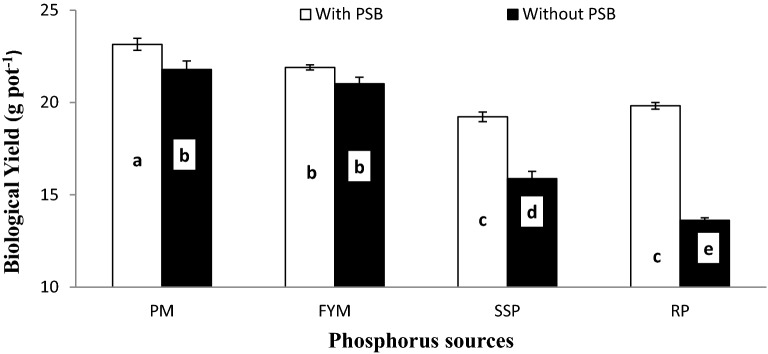
Interactive effect of P sources and PSB on wheat biological yield (g pot^−1^). Graph bars having different letters are significantly different at α = 0.05. Errors bar represent standard error for the mean of three values.

**Figure 9:**
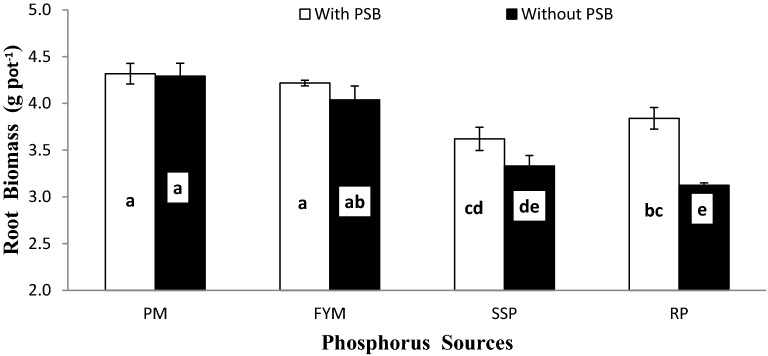
Interactive effect of PSB and P sources on root biomass (g). Graph bars having different letters are significantly different at α = 0.05. Errors bar represent standard error for the mean of three values.

Wheat grain yield was also considerably affected by lime × P sources (L × P) as presented in Fig. 7. The L × P interaction showed a similar pattern for GY. The grain yield significantly decreased with addition of lime into soil at all P sources. Organic sources (PM and FYM) performed better than mineral sources (SSP and RP). There was no significant difference among the response of PM and FYM, and SSP and RP for GY regardless of soil lime concentration. Soil treated with organic sources at 15% lime significantly improved GY over those treated with mineral sources at 10% lime. Wheat BY and RDW were also notably affected by inoculum × P sources (I × PS) as presented in Figs. 8 and 9, respectively. Generally, seed inoculation with PSB markedly improved all mentioned traits over un-inoculated. The response of inoculation was more prominent in mineral (SSP and RP) sources than organic (PM and FYM). In either case, organic sources performed potentially better than mineral sources. There was no difference between inoculated and un-inoculated treatments for PM and FYM in all traits except BY where inoculated PM produced significantly more biomass than un-inoculated. Except biological yield where PM + PSB produced more biomass than FYM + PSB while for the rest of the mentioned traits the performance of PM and FYM were statistically similar irrespective of the inoculation. There was no significant difference among SSP and RP with inoculation in BY and RDW but without inoculation SSP produced more BY than RP whereas, for RDW their differences were un-noticeable.

### Wheat P concentration, uptake and post-harvest soil Olsen P

Effects of PSB, P sources and soil calcification on wheat P concentration (PPC), uptake (PPU) and post-harvest soil Olsen P (PSP) are presented in Table 5. Inoculation, liming and P sources distinctly affected all mentioned traits. Inoculation with PSB increased PPC (7.1%), PPU (24.3%) and PSP (3.4%) over un-inoculated control. Poultry manure proved to be the most potential source for improving these traits and its effect was statistically similar to FYM for PPC and PSP. Rock phosphate performed poorer than the rest of P sources for these traits however its effect was similar to SSP for PSP. Liming adversely affected PPC, PPU and PSP and a gradual decrease in these was observed with increasing lime content from control to 20%. Addition of lime at the rate of 10, 15 and 20% declined PPC, PPU and PSP by 4.1, 21.5 and 36.1% (PPC), 10.7, 30.7 and 56.7% (PPU) and 6.7, 13.6 and 23.6% (PSP), respectively.

**Table 5:** Role of PSB and P sources in improving wheat P concentration, uptake and post-harvest Olsen P content in soil under varying lime levels.

Inoculation	Plant P (%)	P uptake (mg pot^−1^)	Post-harvest soil Olsen P (mg kg^−1^)
Without PSB	0.141	26.3	5.93
With PSB	0.151	32.6	6.14
LSD (α = 0.05)	0.0032	0.984	0.090
**P sources**			
SSP	0.140b	25.4c	5.70b
Rock phosphate	0.135c	23.2d	5.58b
FYM	0.153a	33.4b	6.45a
PM	0.156a	35.8a	6.41a
LSD (α = 0.05)	0.0047	1.391	0.127
**Lime (%)**			
Control (4.78%)	0.172a	35.8a	6.78a
10	0.165b	33.4b	6.33b
15	0.135c	27.1c	5.86c
20	0.110d	16.9d	5.18d
LSD (α = 0.05)	0.0047	1.391	0.127
**Interaction**			
L × I	ns	Figure 10**	ns
L × PS	ns	Figure 11***	Figure 12*
I × PS	Figure 13*	Figure 14***	ns
L × I × PS	ns	ns	ns
CV	5.12	8.20	3.65

PSB, LSD, SSP, FYM, PM and ns denote phosphate solubilizing bacteria, least significant difference, single super phosphate, farmyard manure, poultry manure and non-significant interaction, respectively.

Means with different letter in each column are significantly different at *p* ≤ 0.05.

Single asterisk stands for significant, Double asterisk stands for higly significant, Triple asterisk stands for very higly significant.

**Figure 10:**
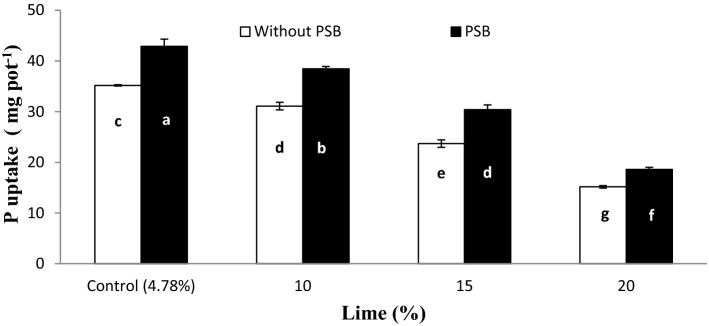
Plant P uptake (mg kg^−1^) affected by lime and PSB**.** Errors bar represent standard error for the mean of three values. Graph bars sharing letters are statistically comparable at *p* ≤ 0.01.

Significant interactive effect of lime and inoculums (L × I) was observed for PPU (Fig. 10). Inoculation with PSB significantly improved PPU over without inoculation at each lime content. PPU decreased with increasing lime content both with and without inoculation pots. Maximum P uptake was recorded for pots amended with control + PSB while the minimum was observed for 20% lime + no PSB. Moreover, the uptake was at par in pots treated with 15% lime with inoculation and 10% lime without inoculation and 10% lime with PSB even performed better than control lime without inoculation.

**Figure 11:**
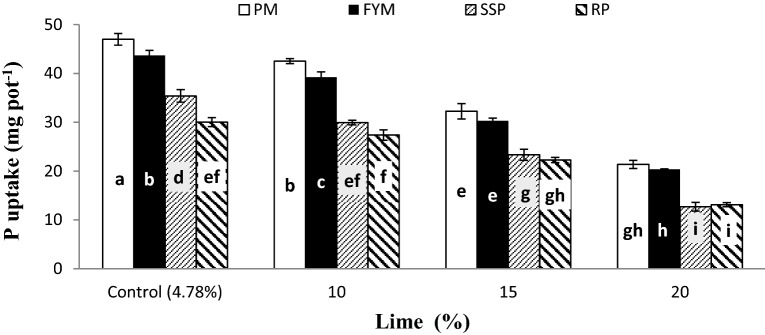
Plant P uptake (mg kg^−1^) as affected by the interaction of P sources and Lime. Graph bars having different letters are significantly different at α = 0.05. Errors bar represent standard error for the mean of three values.

**Figure 12:**
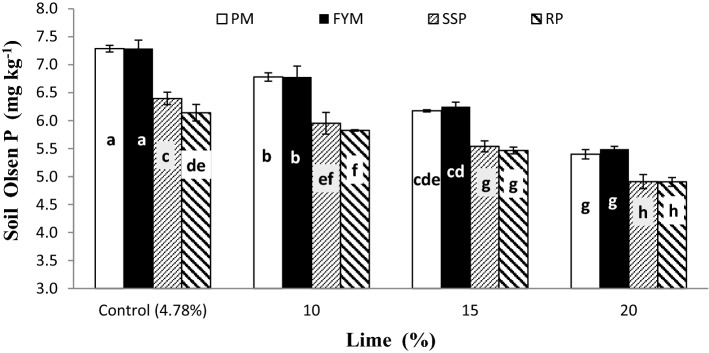
Soil post-harvest P (mg kg^−1^) as affected by P sources and Lime. Graph bars having different letters are significantly different at α = 0.05. Errors bar represent standard error for the mean of three values.

Both PPU and PSP were significantly improved by the interaction of lime and P sources (L × PS) as presented in Figs. 11 and 12, respectively. Application of lime decreased both PPU and PSP regardless of P sources though, FYM and PM played well than RP and SSP. Non-significant variations were noticed between PM and FYM for both PPU and PSP at all lime content excluding control and 10% lime where significantly increased PPU compared to FYM. In non-calcareous soils (4.78% lime) SSP performed statistically better than RP in both PPU and PSP whereas there were no differences in either case among these at the rest of lime. Both PPU and PSP were statistically greater in pots treated with 15% lime and organic sources than 10% lime and mineral sources.

**Figure 13:**
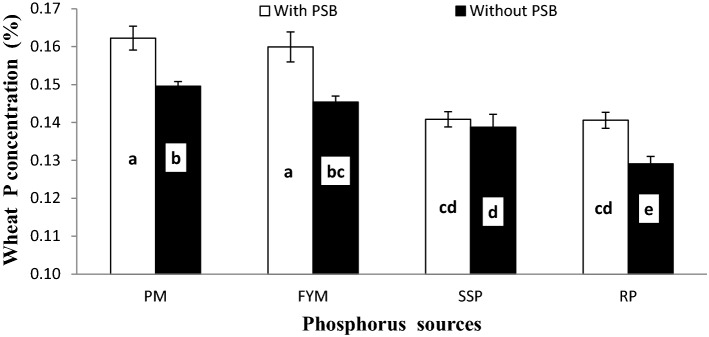
Interactive effect of PSB and P sources on plant P concentration (%).Graph bars having different letters are significantly different at α = 0.05. Errors bar represent standard error for the mean of three values.

**Figure 14:**
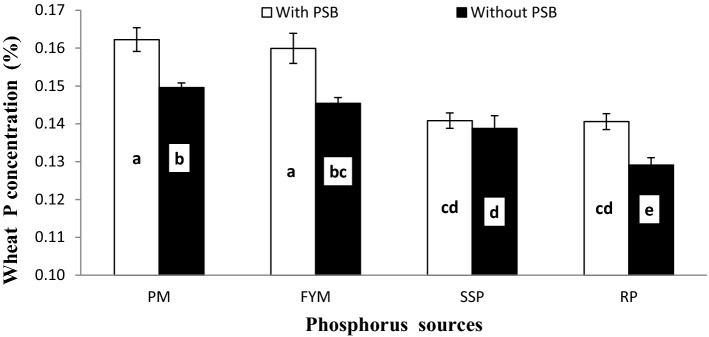
Interactive effect of PSB and P sources on wheat P uptake (mg kg^−1^). Graph bars having different letters are significantly different at α = 0.05. Errors bar represent standard error for the mean of three values.

The response of PPC and PPU was also significant to the interaction of inoculum and P sources (I × PS) as presented in Figs. 13 and 14, respectively. PSB inoculation improved both PPC and PPU over no-inoculation at corresponding P sources except for SSP where there was no effect of inoculation on PPC. Both PPC and PPU were higher in soil treated with Pm and FYM than RP and SSP irrespective of PSB inoculation. Non-significant variation was observed among PM and FYM with and without inoculation for PPC while in case of PPU, PM performed superior than FYM both with and without PSB. Similarly, there were no differences among SSP and RP when inoculated with PSB for PPC and PPU whereas, without inoculation SSP was better than RP. In addition, SSP and RP with PSB responded alike to FYM without PSB in PPC.

### Post-harvest soil EC, pH, organic matter and lime

Findings regarding post-harvest soil EC, pH, soil organic matter (SOM) and lime content as affected by PSB, phosphorus sources and lime are presented in Table 6. There was no statistical difference between inoculated and un-inoculated pots for post-harvest soil EC, pH and lime while, SOM was significantly decreased by about 0.83% in inoculated treatments over control. Similarly, P sources didn’t affect soil EC and lime. A significant decrease in soil pH was observed with the application of organic sources (PM and FYM), however, their effect was not statistically different from SSP. The highest pH was observed at RP which was also similar to SSP. Significant increase in after harvest SOM was observed with addition of P as FYM which was higher than PM and the lowest SOM was observed where P was supplemented through SSP and RP. Liming significantly augmented post-harvest soil EC, pH, SOM and lime content (Table 6). With increasing lime application, all mentioned attributes were gradually increased. Liming increased post-harvest soil EC by 42, 82 and 111, pH by 3, 5 and 6, SOM by 1, 3 and 5 and lime 106,210 and 314% over control (4.78%) at 10, 15 and 20% lime, respectively.

**Table 6:** Post-harvest (wheat) soil EC, pH, organic matter and lime contents as influenced by PSB, phosphorus sources under different lime levels.

Inoculation	Soil EC (dS m^−1^)	Soil pH	Organic matter (%)	Total lime (%)
Without PSB	0.97	8.99	0.843	12.33
With PSB	0.97	8.11	0.836	12.28
LSD (α = 0.05)	ns	0.031	0.0059	ns
**P sources**				
SSP	0.96	8.07ab	0.816c	12.33
Rock phosphate	0.95	8.08a	0.813c	12.31
FYM	0.94	8.05b	0.872a	12.30
PM	0.94	8.05b	0.856b	12.29
LSD (α = 0.05)	ns	0.028	0.0083	ns
**Lime (%)**				
Control (4.78%)	0.59d	7.75d	0.820c	4.77d
10	0.85c	8.05c	0.828c	9.88c
15	1.09b	8.15b	0.848b	14.82b
20	1.27a	8.29a	0.861a	19.76a
LSD (α = 0.05)	0.30	0.028	0.0083	0.058
**Interaction**				
L × I	ns	ns	ns	ns
L × PS	ns	ns	Figure 15***	ns
I × PS	ns	ns	ns	ns
L × I × PS	ns	ns	ns	ns
CV	5.40	0.62	1.73	0.82

PSB, LSD, SSP, FYM, PM and ns denote phosphate solubilizing bacteria, least significant difference, single super phosphate, farmyard manure, poultry manure and non-significant interaction, respectively.

Means with different letter in each column are significantly different at *p* ≤ 0.05.

Triple asterisk stands for very higly significant.

**Figure 15:**
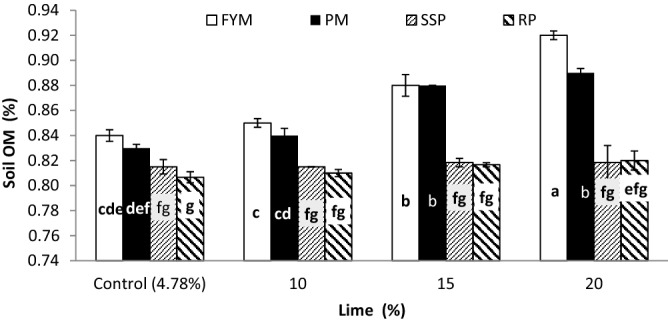
Post-harvest Soil organic matter (%) as affected by interaction of P sources and lime. Graph bars having different letters are significantly different at α = 0.05. Errors bar represent standard error for the mean of three values.

None of the interactions were significant for post-harvest soil EC, pH, SOM and lime except lime and P sources (L × PS) which significantly altered post-harvest SOM (Fig. 15). Addition of lime didn’t affect SOM in pots treated with SSP and RP. Pots where P was applied as organic sources like PM and FYM, SOM significantly varied with liming. Maximum SOM was recorded for FYM at 20% lime followed by PM at 20 and 15% which was at par to FYM at 15% lime. SOM responded alike to PM and FYM at control, 10 and 15% lime while at 20% lime FYM significantly improved soil OM over PM. Furthermore, at each lime level the response of SOM was significantly higher to organic sources than mineral sources.

## Discussion

Over use of mineral phosphorus fertilizer is deteriorating the environment, i.e., biodiversity loss. Continuous use of inorganic fertilizers is also depleting the soil organic matter that is directly associated with water infiltration, microbial proliferation and soil fertility i.e., mineralized P. For sustainable management of soil health and achievement of optimum crop productivity use of organic amendments and rhizobacteria are prime in importance. Organic amendments not only improve soil physical and chemical attributes but also facilitate in microbial proliferation which is beneficial for crop productivity^24^. That’s why for sustainable management of soil nutrients especially phosphorus we examined the potential of PSB under different P supplements in improving wheat yield, P availability and soil properties in artificially calcified soils. Researchers reported *Aspergillus, Bacillus* (*B. subtilis*, *B. polymyxa, B. sircalmous, Bacillus megaterium* and *B. circulans*), *Penicillium, Enterobacter, Pseudomonas and Rhizobium* as the most efficient P solubilizer and could be used as the main strains of PSB^36–38^. We also observed that PSB inoculation improved wheat growth, yield, soil and plant P concentration, and uptake and decreased post-harvest soil organic matter and pH over un-inoculated control. The findings of Tawaraya et al.^39^ supported our results regarding the yield improvement via PSB inoculation in agricultural crops. In this study PSB × lime demonstrated that, PSB inoculation nullified antagonistic effect of liming on plant growth and soil and plant P nutrition which further confirms the findings of Islam and Hossain^40^ who reported enhanced Psolubilizationand improved crop P nutrition in various crops by PSB in calcareous soils. Presence of PSB modified the soil physio-chemical properties which facilitate in solubilization of Fixed/immobilized P in soil. We noticed improvement in germination with PSB inoculation which is in conformity to Amruthesh et al.^41^ who observed increase in germination and seedling vigor of different crop plants with PSB inoculation. Betterment of plant height and tillers per pot by PSB in our study are in confirmation to Kumar et al.^42^ who reported significant increase in plant height and plant density by *Azotobacter chroococcum* inoculation in sorghum. This could be ascribed to better absorption of nutrients, mainly P due to change in soil pH through secretions of organic acid and phosphatase enzyme activity. Rhizobacteria secretes growth regulators, i.e., IAA that significantly enhance root surface area, adventitious and lateral root length. This increase in root length is mainly attributes as improvement in the cell division especially in hypocotyls and less accumulation of dead cell in the cortex region of root^43,44^. In current study, wheat grain, straw and biological yield were improved with PSB inoculation regardless of the P sources used however, the increase was relatively more in pots amended with PM and FYM than SSP and RP. Dwivedi et al.^45^ who observed increase in wheat yield with seed inoculation of PSB. According to Saad and Hammad^46^ application of PSB with calcium superphosphate resulted in maximum grain yield of wheat. Similarly, it is also confirmed by Islam and Hossain^40^ who observed maximum biological yield of wheat crop at rock phosphate inoculated with P solubilizing fungi like *Aspergillus niger* and *Pseudomonas titrinum*. Increase in biological yield of sorghum, maize and rice with PSB inoculation in non-calcareous soils have also been observed by Chabot and Antoun^47^ and Kundu et al.^48^. As like our findings, Kumar et al.^42^ obtained enhanced wheat straw yield by PSB inculcation. According to Sharma et al.^49^ PSB can improve soil productivity via the syntheses of beneficial metabolites, such as, antibiotics, phyto-hormones and siderophores. Afzal et al.^50^ obtained better seed P concentration, tillers, grain and biological yields of wheat. One of the possible reasons for mentioned improvement may be their acting as PGPR. According to Jalili et al.^51^ PSB may improve crop nutrition and growth through production of auxin, ACC-deaminase, root colonization, P solubilization, chitinase activity, siderophores production, and antibiotic production^46^.

We found that, PSB inoculation also significantly increased P availability regardless of whatever P sources was used. Sharma and Prasad^52^ and Vyas and Gulati^53^ also found enhanced cereal growth and nutrition due to PSB inoculation with and without fertilizers application. Our findings are in agreement to Mukherjee and Rai^54^ who also observed increased P uptake in wheat and cotton^55^ due to PSB inoculation over without PSB pots. This improvement in crop nutrition and yield could probably be due to the production of phytohormones^52^ and organic acids such as, acetic, citric gluconic, lactic, isovaleric, 2-ketogluconic, isobutyric, oxalic acid by PSB^56,57^ which acidify soil and enhance P availability to crop. It was also evident from the interaction of PS × I that RP performed comparable to SSP when inoculated with PSB for most of the agronomic and soil parameters. It could be because of enhanced P release from RP by PSB due to production of organic acids which enhances P solubalization^58^. It can be attributed to reduction of soil pH by PSB through the release of organic acids which increase P solubalization from RP as cited by Banik and Dey^59^. The PSB promoted soil acidification and enhanced crop P concentration and uptake over without PSB treatments irrespective of lime levels and P sources however, the organic sources were superior to mineral sources. This could be attributed to chelation of cations like (Al, Fe and Ca) and decreases of soil pH by the hydroxyl and carboxyl groups of acids produced by PSB and organic manures^60^. Ekin^61^ and Zabihi et al.^62^ indicated that PSB inoculation increases the efficiency of P fertilizers. Gulati et al.^63^ reported improved crop P nutrition by seed inoculation with PSB. Improvement in root biomass due to PSB inoculation may probably be due to syntheses of growth regulators at the root interface by PSB, which stimulates root development and promotes water and nutrients absorption by plants from the soil^64^. Our results demonstrate that, P must be applied as organic sources like PM and FYM both to calcareous and non-calcareous soils for lowering soil pH and improving soil OM contents. Sharma and Prasad^52^ also observed enhanced P availability with PSB inoculation which was further advanced with addition of crop residues. It may be attributed to the improvement of soil physical conditions, microbial growth, extra nutrient supplementation and soil acidification by organic sources^65^. The relative inferior performance of mineral sources may be due to quick fixation of available P form these sources which renders its availability to plant as reported by Biswas^66^. In our case organic manures (PM and FYM) application decreased soil pH, improved wheat P concentration, and uptake and nullified antagonistic effect of lime due to solubilization of native nutrients as also reported by Mitra et al.^67^. Similar findings were also reported by Dwivedi et al.^45^ in non-calcareous soils. The acidification of soil by PM and FYM may be attributed to release of organic acids during the process of their decomposition. Mitra et al.^67^ and Laxminarayana^68^ also found similar increase in P availability and uptake by integrated nutrient management in sun hemp.

Generally, liming of alkaline soils adversely affected overall soil and plant parameters; however organic manures and PSB were capable of neutralizing/minimizing this harmfulness up to some extent. It could be due to precipitation of available P with Ca^+2^ ion of lime in alkaline soil which render P availability in soil and its uptake by plants^65^. Sanyal and De Datta^69^ reported that P precipitates as a range of mono-(CaHPO_4_), di- and tri-Ca phosphates [e.g. Ca_3_(PO_4_)_2_] and hydrates in alkaline calcareous soils. In contrast to our finding Briedis et al.^70^ and Bronick and Lal^71^ reported that liming of an acid soils neutralize soil pH, improve root, shoot and soil organic carbon storage. We noticed that liming increased post-harvest SOM. The PSB may substitute the costly mineral fertilizers by natural, economical and eco-friendly P sources like phosphate rocks. PSB may also reduce the exogenous application of costly phosphatic fertilizers by enhancing fertilizers use efficiency through effective utilization of insoluble reserved phosphorus in calcareous alkaline soils.

## Conclusions

The inoculation (PSB) was effective in improving crop growth, P nutrition and soil acidification when compared to un-inoculated control (without PSB), irrespective of P sources and varying level of lime. Individually, liming antagonized plant growth, P availability and induced soil salinity and alkalinity that’s why, non-calcareous soil is the best soil for optimum crop growth. However, PSB inoculation along with manures (PM and FYM) application potentially minimized the adverse effects of liming over mentioned traits. Solely, organic P supplements (PM and FYM) performed significantly better than mineral supplements (SSP and RP) in advancing wheat growth and soil condition but when mineral sources were inoculated with PSB its performance were mostly comparable to organic sources. Rock Phosphate with PSB has been shown as effective as sole SSP. Therefore, P application as organic manures in conjugation to PSB inoculation can be an environmental friendly and sustainable approach for improving plant growth and properties of calcareous soils. Furthermore, RP can be used as potential substitute to SSP if inoculated with PSB. It is highly recommended that more research and investigation can be done for other crops with the combine application of organic manures and PSB for sustainable agriculture. However, these findings shall be verified under diverse agro-climatic conditions on variety of crops before formulating large scale recommendations.

## Materials and methods

### Soil description

A surface (0–20 cm) soil (Gulyana soil series) was obtained from field under wheat–maize cultivation at Agricultural Research Station, Bajabamkhel, District Swabi, KPK-Pakistan (34° 7′ 12′′ North and 72° 28′ 20′′ East). The soil was shade dried and sieved (2 mm). It was alkaline (pH 7.56) and non-saline (0.76 d Sm^−1^), non-calcareous (4.78% lime) in nature and silt loam in texture. The soil was low in organic matter (0.82%), and deficient in Olsen extractable P (5.28 mg kg^−1^), K (78 mg kg^−1^) and total N (0.08 g kg^−1^) as shown in Table 7. According to the USDA classification system the soil was classified as Inceptisols soil with Ochric surface horizon.

**Table 7:** Physico-chemical properties of soil used in experiment before cultivation.

Property	Quantity
Bulk density	1.24 g cm^−3^
Textural class	Silt loam
Soil pH	7.56
EC_*e* 1:2_	0.76 dSm^−1^
CEC	36.1 cmole kg^−1^
Total lime	4.78%
Organic matter	8.2 g kg^−1^
Total nitrogen	0.08 g kg^−1^
Olsen P	5.28 mg kg^−1^
Potassium (K)	78 mg kg^−1^

### Experimental materials

The poultry and farm yard manures were purchased from nearby dairy and poultry farms, respectively, and were analyzed for their nitrogen, phosphorus and potassium (NPK) concentration (Table 8). The powdered RP was acquired from Nuclear Institute of Food and Agriculture (NIFA), Peshawar and was analysed for its P concentration. The PSB was obtained from National Agriculture Research Center and was examined for its composition, population and plant growth promoting rhizobacteria (PGPR) characterization.

**Table 8:** NPK composition of P sources.

Source	Total N	P	K
	(%)		
RP	–	17.1	–
PM	2.26	1.4	1.28
FYM	1.35	0.88	1.03

RP, PM and FYM stands for rock phosphate, poultry manure and farm yard manure.

### Characterization of used PSB

The bacterial composition of the inoculum was examined using Bergeys manual of systematic bacteriology^72–74^ on modified Pikovskaya’s agar medium amended with Ca_3_(PO_4_)_2_ as an insoluble P. Phosphate solubilization^75^ and P content in the culture supernatant was measured by the procedure of Nelson and Sommrs^76^. The PSB were tested for alkaline phosphatase activity^77^, siderophores^78^, and IAA and organic acid production^79^.

### Experimental procedures

This pot experiment was consisted of two forms of inoculation (without PSB and with PSB), four types of P supplements [poultry manure (PM), farm yard manure (FYM), single super phosphate (SSP) and rock phosphate (RP)] applied @ of 45 mg P_2_O_5_ kg^−1^ soil under varying (4) lime (4.78, 10, 15 and 20%) content making 32 treatments, in pots containing 7 kg soil (including natural/added lime). Factorial (3) complete randomized design (CRD) with three replications was used. Lime was applied 30 days before sowing while, P sources and PSB were supplemented at the time of sowing. Phosphorus was supplemented as SSP, RP, PM and FYM at the rate of 45 mg P_2_O_5_ kg^−1^ on the basis of P contents (Table 2) as per proposed treatments. Urea and SOP were applied in solution form to all pots at the rate of 60 mg N kg^−1^ and 30 mg K_2_O kg^−1^ soil (including N and K added from organic sources) as a basal dose. PSB inoculum containing 1.75 × 10^8^, cfu of PSB g^−1^ (wet weight), was applied as seed inoculation @ of 2 kg PSB inoculum per 120 kg seed^80^. Post inoculation PSB per seeds were 1.36 × 10^5^ CFU^81^. Ten seeds were sown in each pot (30 cm diameter) which was later thinned to 6 plants. The pots were sited in open atmosphere followed by periodical randomization. The pots were retained at around 60% of field capacity (FC) by irrigation with tap water on daily basis as per procedure Wu et al.^82^. The pots were kept outside in a netted enclosure so that the pots would reflect the outside air temperature and the environment. The cultural practices recommended for pot experiments were followed during the study.

### Agronomic parameters of wheat

Days to emergence for wheat were calculated by counting the days taken from date of sowing till 75% emergence occurred in all pots. Emergence per pot was taken by totaling the seedlings emerged in each pot after germination. Tillers plant^−1^ were recorded at maturity by counting the tillers of two randomly selected plants in each pot and then averaged. Plant height (cm) was calculated by measuring height of two randomly selected plants from base to the tip of the plants excluding awns with the help of meter rod in each pot and then averaged. Days to maturity were calculated by counting the number of days from date of planting to 75% physiological maturity. Grains spike^−1^ were counted by threshing two randomly selected spikes from each pot and averaged. A sample of hundred grains was taken randomly from each pot and weighed using a sensitive electronic balance to record 100 grains weight. For biological yield**,** all plants of each pot were harvested and allowed to dry under sun for five days and then weighed. Grain yield was recorded by weighing the grains obtained from whole pot after threshing and cleaning. The roots collected from each pot were washed, dried and their biomass was recorded via electronic balance.

### Soil and plant analysis

Soil pH and EC in 1: 5 soil water saturation extract were measured by the procedure of Thomas^83^ and Rhoades^84^, correspondingly. N and K contents in soil were quantified by Kjeldhal method^85^ and Ryan et al.^86^, respectively. Olsen NaHCO_3_ protocol was adopted for determination of P in soil^87^. The lime content was calculated by titration method^88^, texture by Bahadur et al.^89^ while, soil OM content was quantified by the method of Nelson and Sommer^90^. Polemio and Rhoades^88^ protocol was used for determination of soil cation exchange capacity. Acid digestion method used by Richards^91^ was applied for determination of plant P concentration. P uptake was taken as a product of the plant biomass and its respective concentration in each pot.

### Statistical analysis

For PGPR characterization descriptive statistics was applied for calculating standard error. Data regarding plant parameters and post-harvest soil properties were subjected to Fisher’s (F) test for analysis of variance^92^ by using Statistix 8.1. The results were further run for least significant difference (LSD) test to find the difference among the means.

### Complies with international, national and/or institutional guidelines

Experimental research and field studies on plants (either cultivated or wild), comply with relevant institutional, national, and international guidelines and legislation. Experimental studies were carried out in accordance with relevant institutional, national or international guidelines or regulation.

### Permissions or licenses

The experiment was started, after taking permission from The University of Agriculture, Peshawar, Khyber Pakhtunkhwa, Pakistan.

### Identification of the plant material

Before collection, the plant was identified by Dr. Hanif Khan (Taxonomist), using the standard protocol at the Department of Soil Science, Agricultural University, Peshawar, Pakistan.

### Ethics approval and consent to participate

We all declare that manuscripts reporting studies do not involve any human participants, human data, or human tissue. So, it is not applicable.

## Data Availability

The datasets generated and/or analysed during the current study are not publicly available, but are available from the corresponding author on reasonable request.
